# Shoulder pain: Is the outcome of manual therapy, acupuncture and electrotherapy different for people with high compared to low pain self-efficacy? An analysis of effect moderation

**DOI:** 10.1177/17585732221105562

**Published:** 2022-06-20

**Authors:** Bradley Rugg, Mizanur Khondoker, Rachel Chester

**Affiliations:** 1 School of Health Sciences, Faculty of Medicine and Health Sciences, 6106 University of East Anglia, Norwich, Norfolk NR4 7TJ, UK; 2 Physiotherapy Department, 40424 The Clementine Churchill Hospital, Sudbury Hill, Harrow, HA1 3RX, UK; 3 Norwich Medical School, Faculty of Medicine and Health Sciences, 6106 University of East Anglia, Norwich, Norfolk NR4 7TJ, UK

**Keywords:** Shoulder, physiotherapy, moderator, treatment, pain self-efficacy

## Abstract

**Background:**

High baseline pain self-efficacy (PSE) predicts a better outcome for people attending physiotherapy for musculoskeletal shoulder pain. A potential contributing factor is that PSE moderates the relationship between some treatment modalities and outcome. Our aim was to investigate whether there is a difference in outcome between participants with high compared to low PSE receiving manual therapy, acupuncture, and electrotherapy.

**Methods:**

Participants were stratified into high or low baseline (i) PSE, (ii) shoulder pain and disability index (SPADI), and (iii) did or did not receive the treatment. Whether the effect of treatment differs for people with high compared to low PSE was assessed using the 95% confidence interval of the difference of difference (DoD) at a 5% significance level (*p* < 0.05).

**Results:**

Six-month SPADI scores were consistently lower (less pain and disability) for those who did not receive passive treatments compared to those who did (statistically significant less pain and disability in 7 of 24 models). However, DoD was statistically insignificant.

**Conclusion:**

PSE did not moderate the relationship between treatment and outcome. However, participants who received passive treatment experienced equal or more pain and disability at 6 months compared to those who did not. Results are subject to confounding by indication but do indicate the need for further appropriately designed research.

**Level of Evidence:**

Level of evidence II-b.

## Introduction

Shoulder pain is one of the most common musculoskeletal disorders^1^ with up to 3% of adults presenting with new shoulder pain annually.^2,3^ Of those who visit their General Practitioner, 48% require repeated visits due to ongoing pain and disability.^4,5^ For people with a wide range of musculoskeletal problems, including shoulder pain, evidence from systematic reviews consistently shows that self-management strategies, including exercises advised by physiotherapists, effectively reduce pain, increase function and improve quality of life.^6^ Many physiotherapists augment these with passive treatment modalities such as manual therapy, acupuncture and electrotherapy,^7^ the evidence for which is mixed.

Pain self-efficacy (PSE) at the start of treatment is a significant predictor of outcome for people attending physiotherapy for the management of musculoskeletal shoulder pain.^8–10^ PSE is the strength of confidence or belief a person has in their ability to complete tasks and reach a desired outcome despite their shoulder pain.^11^ Our Classification and Regression Tree (CART) analysis demonstrated that when using the pain subscale of the shoulder pain and disability index (SPADI) as an outcome of physiotherapy at six-month follow-up, with the exception of baseline SPADI scores, PSE was the most important predictor of outcome.^9^ This was also true for participants with high baseline SPADI disability. In summary, whilst high baseline pain and disability were associated with a poor outcome, concomitant high PSE often changed this to a good predicted outcome. Conversely, whilst a low baseline pain SPADI subscore was associated with a good outcome, concomitant low PSE often changed this to a poor predicted outcome.

The mechanisms by which PSE is associated with outcome are uncertain. One possibility is that PSE moderates the relationship between some treatment modalities and outcome.^12,13^ Ninety-nine per cent of participants in our study received advice and exercises as a treatment modality. Sixty-four per cent of those providing six months follow-up data received manual therapy, acupuncture, or electrotherapy. The objective of this analysis was to investigate whether there is a difference in outcome for people receiving manual therapy, acupuncture, or electrotherapy, based on their baseline PSE.

## Methods

### Study population

Data were available for 1030 participants recruited to a multicentre longitudinal cohort study in the East of England between November 2011 and October 2013. The protocol has been published in detail and is summarized here,^8,14^ relevant to the aims of this report. Study approval was obtained from the National Research Ethics Service, East of England-Norfolk, UK in July 2011 (reference 11/EE/0212, protocol number R18870). Participants were informed of all their rights, including the right to leave the study at any stage without the need to provide a reason and that this would not affect the quality of care they would receive. Participants provided written informed consent at their first physiotherapy appointment.

Participants were attending physiotherapy for the management of musculoskeletal shoulder pain in primary or secondary care. Participants aged 18 or over were eligible if their shoulder pain, of any duration, was reproducible on active or passive movement of the shoulder. Exclusion criteria included reproduction of shoulder pain on spinal rather than shoulder movement, fracture, traumatic dislocation, or surgery of the affected shoulder in the previous five years, complex regional pain syndrome, radiculopathy, or a systemic source of pain.

### Baseline and outcome measurements

The SPADI^15,16^ is a joint-specific, patient-rated questionnaire designed to measure shoulder pain and disability. SPADI is able to differentiate between patients with high and low levels of shoulder pain, and clinical improvers and non-improvers undergoing physiotherapy treatment for musculoskeletal shoulder pain at six months.^17^ The SPADI has been mapped to body function and/or activity components within the ICF^18^ has high internal consistency (Cronbach's alpha 0.86–0.96),^19^ high responsiveness,^17,19^ test–retest reliability coefficients ranging from 0.84 to 0.90 for conservative intervention^19^ and minimal floor and ceiling effects.^19,20^

The SPADI was completed at baseline, prior to participants’ first physiotherapy appointment and via a postal questionnaire six months later. It includes 13 items, five comprising a pain subscale and eight a disability subscale, each of which are scored from 0 to 10, 0 representing no pain or disability, and 10 representing the worst pain imaginable or so difficult it requires help. The pain and disability subscale scores and total SPADI score are expressed as a percentage where 100% represents maximum pain or disability.

PSE was measured prior to the patient's first physiotherapy appointment using the pain self efficacy questionnaire.^21^ This 10-item questionnaire has excellent validity, reliability, and responsiveness in populations with musculoskeletal disorders.^22^ Items are rated 0 to 6, 0 representing minimum PSE and 6 representing maximum PSE. The maximum score of 60 represents a high PSE.

### Treatment data

Details of treatments delivered by physiotherapists were recorded on a custom-designed clinical record form, physiotherapists ticking “Yes” or “No” boxes for different options. Treatment was labelled using 3 categories, 2 of which were subcategories of the first

- “Any passive treatment” – any form of manual therapy and/or acupuncture and/or electrotherapy.
- “Any manual therapy” – shoulder or spine joint mobilisations, deep transverse frictions, capsular stretches, trigger point therapy, muscle facilitation, or other techniques listed by the treating physiotherapist.
- “Spinal/shoulder joint mobilisation” – for example, Maitland, Kaltenborn or Mulligan techniques.

To be categorised, treatment must have been delivered by the physiotherapist at least once and may have been delivered in conjunction with other treatments. The frequency of treatment was not considered for this analysis. Though treatment decisions and management were unaffected by study involvement, physiotherapists were directed to adhere to The Chartered Society of Physiotherapy’s evidence-based guidelines for the management of shoulder pain published at that time.^23,24^

### Analysis

**Figure 1.**
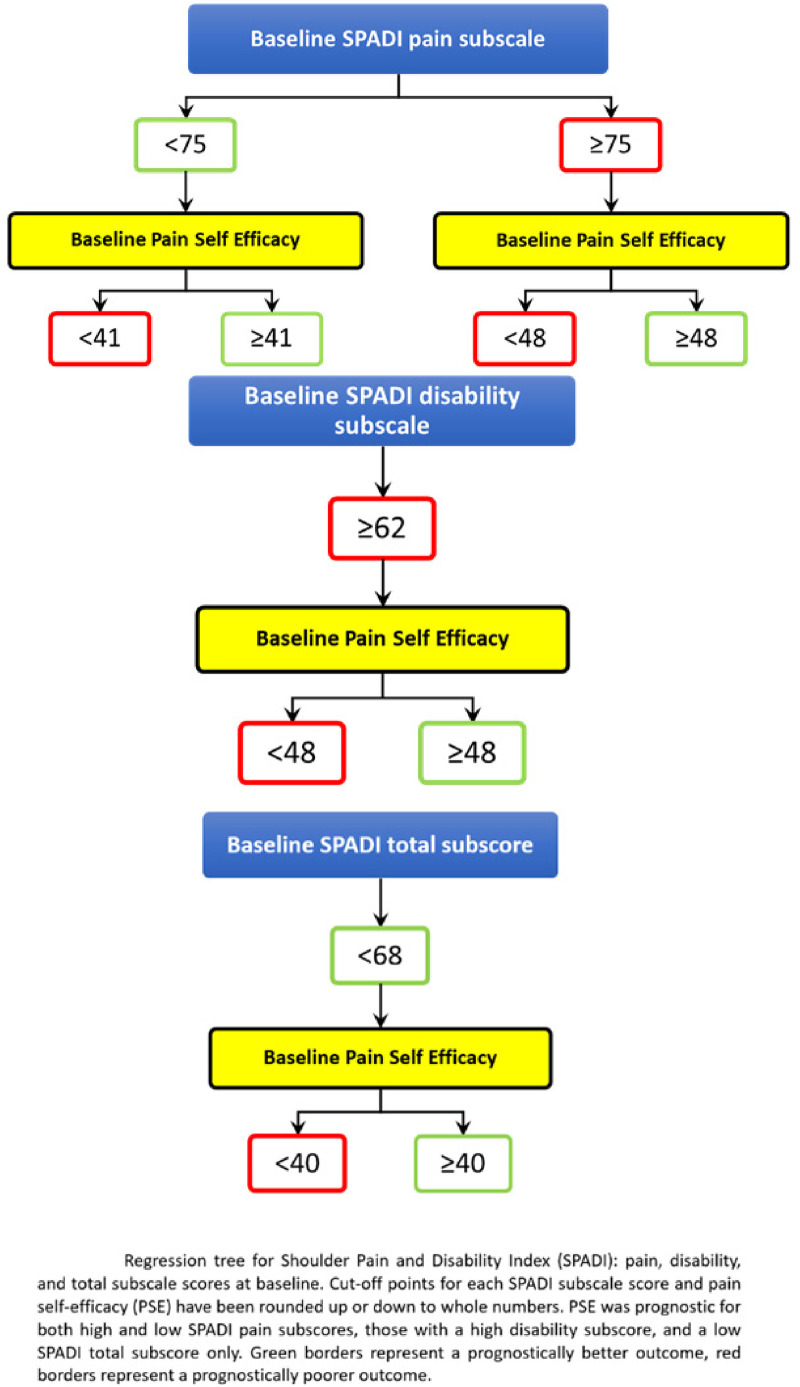
Regression tree for shoulder pain and disability index (SPADI).

At six-month follow-up, our CART analysis used recursive partitioning to produce subgroups as homogeneous as possible with respect to the following outcomes: (i) the SPADI total score and its subgroups, (ii) SPADI pain and (iii) SPADI disability.^9^ All factors statistically associated with outcome (*p* ≤ 0.05) in at least one of the multivariable linear models from our original analysis^8^ were included in the CART analysis. Of 34 baseline factors entered into the CART, only 2 factors predicted outcome: baseline SPADI and baseline PSE. Cut-off points to enable subgrouping and define predictive factors, that is high and low baseline SPADI total and subscores and baseline PSE were computed by the CART (see Figure 1). PSE scores were not predictors of outcome for participants with *low* baseline disability and *high* baseline total SPADI scores, these groups are therefore not described further or included in our analysis. In summary, we performed our analysis for the remaining four groups of participants, defined by their baseline SPADI scores:

- Low (<68) baseline SPADI total score.
- Low (<75) baseline SPADI pain subscore.
- High (≥75) baseline SPADI pain subscore.
- High (≥62) baseline SPADI disability subscore.

Cut-off points, defined by the CART analysis, for high and low PSE scores differed according to the level of baseline pain and disability. High PSE was defined as ≥48 for patient groups with high baseline pain or disability subscores ≥41 for patient groups with a low baseline SPADI pain subscores, and ≥40 for patients with a low total SPADI score (see Figure 1).

### Descriptive statistics

The initial descriptive analysis allowed visual observation and comparison of six-month outcome (mean and standard deviation) for four groups: PSE (high or low) and treatment modality received (yes or no).

### Inferential statistics

Inferential statistics were performed in stages to investigate:

#### Stage 1: does the treatment category have an effect?

For the three treatment categories, four between-group differences represent the effectiveness of the intervention. These are:

- Difference in mean outcome (SPADI, pain or disability) between the high and low PSE groups for participants (i) receiving and (ii) not receiving treatment.
- Difference in mean outcome (SPADI, pain and disability) between participants receiving and not receiving treatment for groups with (i) high and (ii) low PSE.

All mean between-group differences will be presented with 95% confidence intervals (CI) to assess statistical significance at a 5% level (*p* ≤ 0.05).

#### Stage 2: does the treatment effect (if significant) differ by PSE level?

The difference of difference (DoD) value represents the interaction effect between treatment category (did and did not receive) and PSE level (high and low) for each group. The DoD score defines how treatment effect (difference between receiving and not receiving the treatment) differs for those with high and low PSE. That is, a significant DoD value assessed via the 95% CI (the null value 0 being outside the interval) suggests the effectiveness of an intervention differs between participants with high and low PSE (*p* ≤ 0.05).

## Results

**Figure 2.**
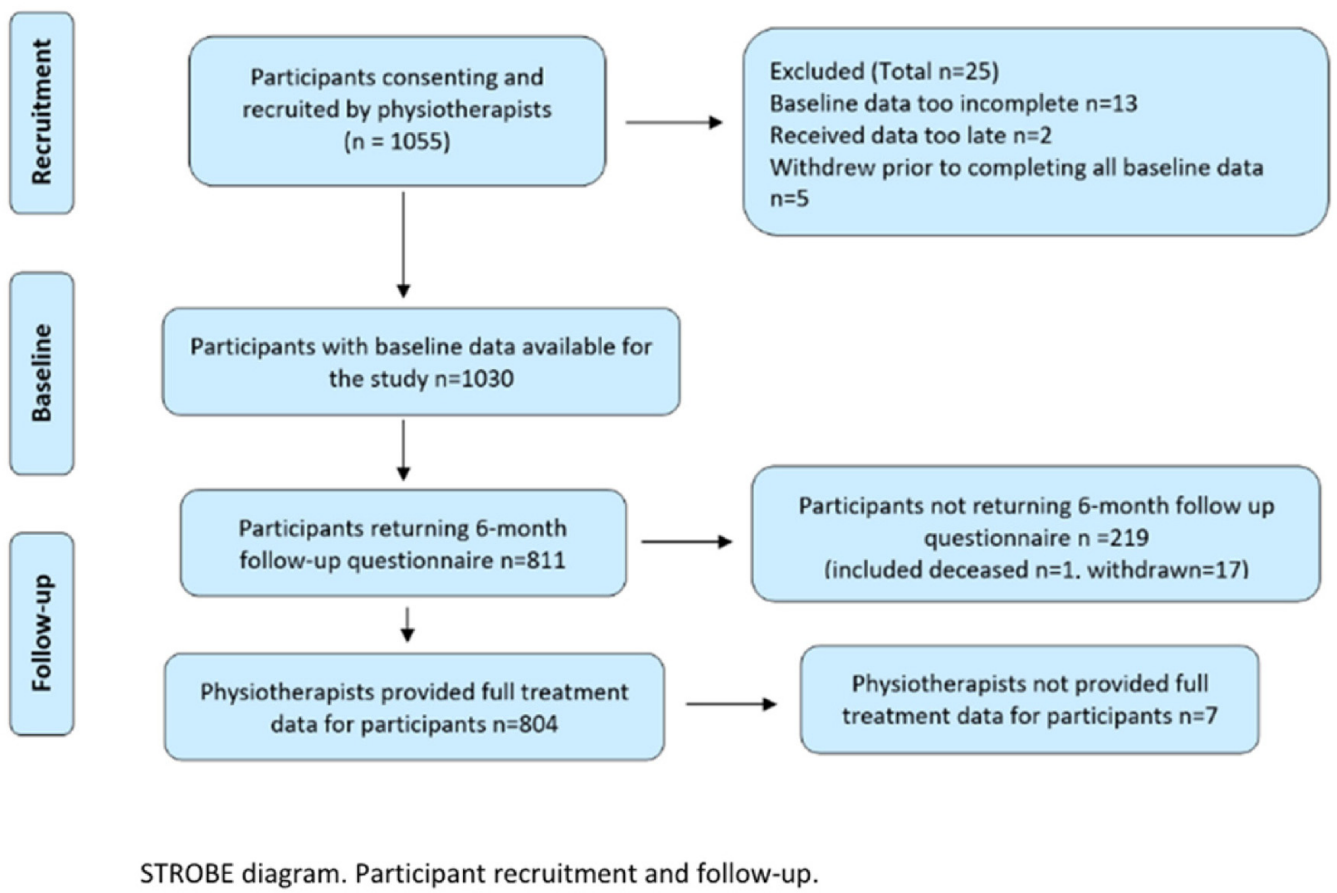
STROBE diagram.

One thousand and fifty-five participants were recruited and consented to the primary study, 1030 of whom were eligible and provided full baseline data. Eight hundred and eleven (79%) participants provided outcome data at six months; physiotherapists provided treatment data for 804 (78%). See the STROBE flow diagram in Figure 2. The mean age of participants available for this analysis was 59 years (SD 14), 362 (45%) of whom were male. Duration of symptoms ranged from 4 days to 7 years.

### Any passive treatment

#### Descriptive statistics

**Figure 3.**
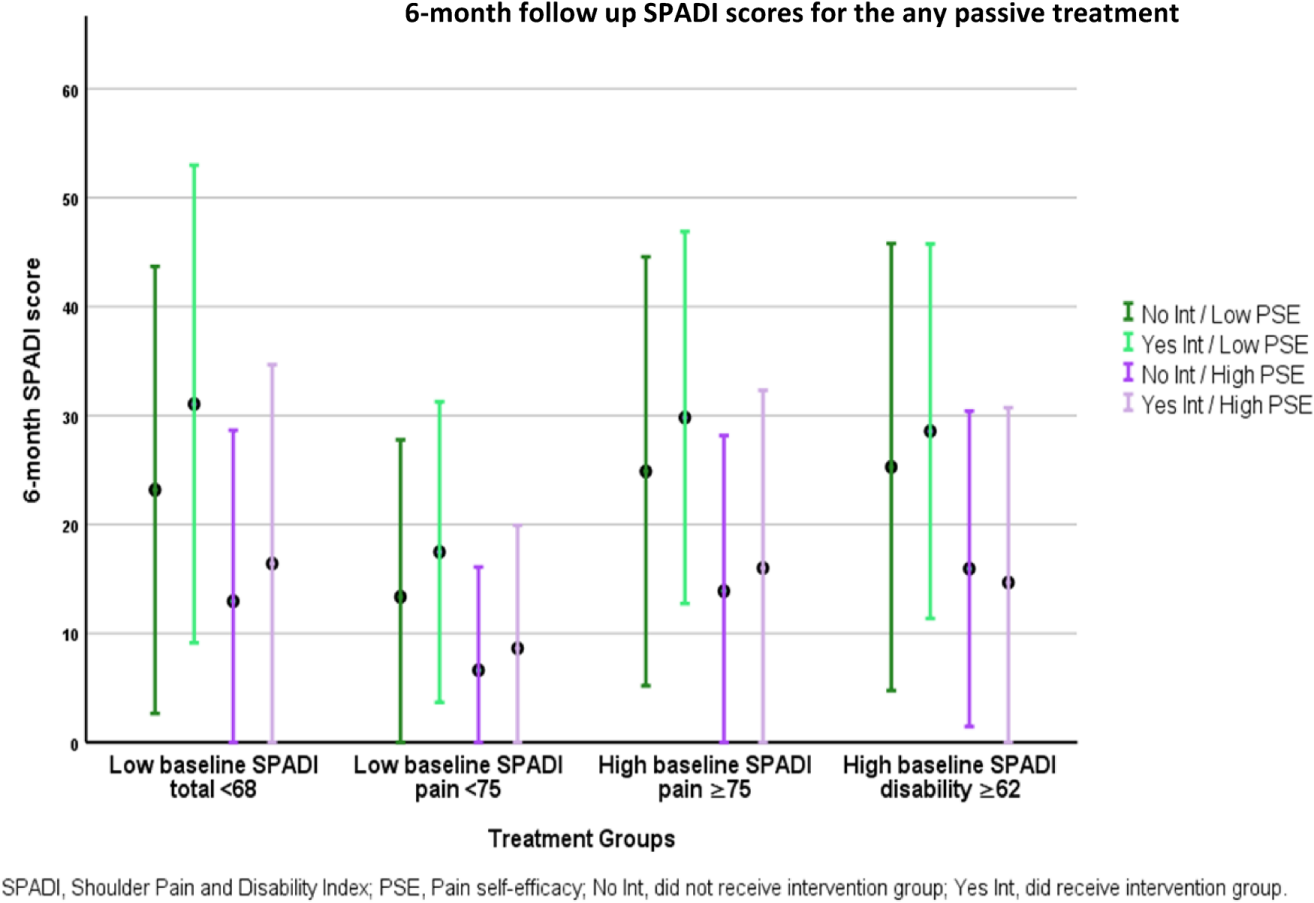
Six-month follow-up shoulder pain and disability index (SPADI) scores for any passive treatment category.

The mean six-month follow-up SPADI scores (total, pain and disability subscores) for those receiving or not receiving any passive treatment, with respect to PSE level are displayed in Figure 3 and Table 1, respectively. The six-month SPADI pain subscore is displayed twice, representing those who had high (≥75) and low (<75) baseline scores, respectively.

**Table 1.** Mean ± SD SPADI subscores at six-month follow-up, differences between treatment and PSE groups, and DoD (interaction) for any passive treatment category.

		High PSE group		Low PSE group	Difference between PSE groups
SPADI subscore		Mean ± SD (*n*)			Mean (95% CI)
Low SPADI total (<68)	Received treatment	16.41 ± 18.26 (300)	31.05 ± 21.92 (95)		−14.65 (−18.89, −10.40)
	Did not receive treatment	12.96 ± 15.69 (182)	23.17 ± 20.51 (47)		−10.21 (−16.11, −4.32)
Difference between treatment groups mean (95% CI)		3.45 (0.06, 6.84)	7.88 (1.46, 14.31)		DoD −4.43 (−11.70, 2.83)
Low SPADI pain (<75)	Received treatment	8.63 ± 11.29 (299)	17.47 ± 13.80 (101)		−8.84 (−11.45, −6.23)
	Did not receive treatment	6.62 ± 9.47 (179)	13.35 ± 14.42 (52)		−6.73 (−10.31, −3.16)
Difference between treatment groups mean (95% CI)		2.02 (−0.13, 4.16)	4.12 (0.25, 7.99)		DoD −2.10 (−6.53, 2.32)
High SPADI pain (≥75)	Received treatment	16.00 ± 16.32 (35)	29.81 ± 17.07 (79)		−13.81 (−20.81, −6.82)
	Did not receive treatment	13.89 ± 14.28 (14)	24.87 ± 19.69 (45)		−10.99 (−21.53, −0.44)
Difference between treatment groups Mean (95% CI)		2.11 (−8.78, 13.01)	4.94 (−1.49, 11.38)		DoD −2.83 (−15.48, 9.83)
High SPADI disability (≥62)	Received treatment	14.67 ± 16.05 (36)	28.56 ± 17.18 (105)		−13.90 (−20.65, −7.14)
	Did not receive treatment	15.93 ± 14.48 (12)	25.27 ± 20.52 (49)		−9.34 (−20.60, 1.92)
Difference between treatment groups mean (95% CI)		−1.27 (−12.92, 10.39)	3.29 (−2.76, 9.34)		DoD −4.56 (−17.69, 8.57)

PSE, pain self-efficacy; SPADI, shoulder pain and disability index; SD, standard deviation; CI, confidence interval; n, number of participants; DoD: difference of difference.

The dark and light green bars on the left of each SPADI subscore represent those with low PSE (see Figure 1 for cut-off points). These subgroups differ only by those who receive and do not receive any passive treatment, the lighter shade representing those who received treatment. The dark and light purple bars on the right of each SPADI subscore represent those with high baseline PSE (see Figure 1 for cut-off points). These subgroups again differ only by those who receive and do not receive any passive treatment, the lighter shade representing those who received treatment.

For all groups with low baseline PSE, irrespective of baseline SPADI, mean six-month SPADI scores are higher (more pain and disability) for those who received treatment (lighter green shaded boxes) compared to those who did not receive treatment (darker green shaded boxes).

The dark and light purple bars on the right of each SPADI subscore represent those with high PSE (see Figure 1 for cut-off points). For groups with high baseline PSE mean six-month scores differed according to baseline SPADI. For groups with low baseline SPADI, all mean six-month SPADI scores are higher (more pain and disability) for those who received treatment (lighter purple shaded boxes) compared to those who did not receive treatment (darker purple shaded boxes). In contrast, for the two groups with high baseline SPADI (more pain and disability), one mean six-month SPADI score is lower (less pain and disability) for those who received treatment compared to those who did not.

#### Inferential statistics

The treatment effect:

1. The mean difference in six-month SPADI between groups with high and low PSE, for participants receiving and not receiving treatment was statistically significant in 7 of 8 analyses; participants with higher baseline PSE had lower SPADI scores (less pain and disability) at six month follow up. See final column, middle rows of Table 1.
2. The mean difference in six-month SPADI between groups receiving and not receiving treatment was statistically significant in 3 of the 4 analyses for participants with low baseline SPADI scores. That is, participants receiving treatment had higher six-month SPADI scores (higher pain and disability). This was for participants with low baseline SPADI total scores with both high PSE (mean change, 3.45; 95% CI: 0.06, 6.84) and low PSE (mean change, 7.88; 95% CI: 1.46, 14.31), and those with low baseline SPADI pain and low PSE (mean change, 4.12; 95% CI: 0.25, 7.99). There was no significant difference in outcome for participants with high baseline SPADI.

### Interaction between treatment and PSE group

DoD (whether the effect of the intervention varies by PSE level) was statistically insignificant for all subscores; low SPADI total (DoD, −4.43; 95% CI: −11.70, 2.83), low SPADI pain (DoD, −2.10; 95% CI: −6.53, 2.32), high SPADI pain (DoD, −2.83; 95% CI: −15.48, 9.83), high SPADI disability (DoD, −4.56; 95% CI: −17.69, 8.57).

Similar results were found for our subgroup analysis of ‘Any Manual Therapy’ and ‘Spinal/shoulder Joint Mobilisations’. For 4 of 16 analyses, participants receiving these treatments had significantly poorer mean outcomes. However, there was no interaction with PSE. See online Supplemental Files S1–4.

## Discussion

The primary objective of this study was to investigate whether there is a difference in outcome for people with high and low PSE with respect to receiving or not receiving passive treatment interventions. This was in a multicentre cohort of patients receiving physiotherapy for musculoskeletal shoulder pain.

As reported in our previous publications, participants with high PSE consistently had significantly better outcomes than those with lower PSE. Except for participants with high baseline PSE and high baseline disability in the passive treatment category (for whom numbers were small and for whom results were more equivocal), participants who received passive treatment in any of the analysed categories had poorer mean outcomes. This was statistically significant in 7 of 24 of our analyses. However, there was no interaction effect which suggests that PSE did not moderate the relationship between manual therapy, acupuncture, electrotherapy, and outcome.

### Comparison with other studies

To our knowledge, this is the first study investigating PSE as a treatment effect moderator for physiotherapy interventions. Much of the related research investigates PSE as a prognostic factor.^8^ For example, for lower back pain^25^ or as a potential protective factor for pain development^26^ and chronicity.^27^ PSE has also been reported to mediate the relationship between depressive symptoms and pain severity for those with lower back pain.^28^

### Strengths and limitations

This secondary analysis of over 800 participants is the first to investigate whether the outcome following passive physiotherapy modalities is moderated by the level of baseline PSE. Participants were selected from a multicentre prospective cohort study in 11 National Health Service trusts and social enterprises in the East of England and included general practitioner sites, primary and secondary care hospital sites, and are therefore likely to represent the wide range of patients seen by physiotherapists in these sectors.

A limitation of our analyses is that there was considerable variation in the number of participants within treatment and PSE groups, ranging from *n* = 12 to *n* = 300. This may have affected the statistical power of the results. Indeed, our equivocal results were for the categories with the lowest number of participants. Additionally, some treatment interventions were not used in isolation but were combined, some were offered on just one occasion, sometimes on multiple occasions. Given the variability in selected treatments used by physiotherapists, specific inferences based on passive treatments using our data is unclear.

Although the primary objective of this report was to investigate PSE as a moderator between treatment effect and outcome, our results suggest no treatment effect is present. In the absence of a treatment effect, no interaction effects can be explored.

Given the study design, the results of our analysis should be viewed with caution. The decision to explore associations between PSE and treatment was not decided a priori to data collection. Participants were not randomly allocated to treatment and did not therefore have an equal chance of receiving or not receiving treatment. Confounding by indication arises when decisions to use a specific treatment are influenced by other factors and are not controlled in observational studies. Treatment selection is complex and based upon multiple factors, some of which may be unknown but associated with treatment outcome. Despite these limitations, the potential clinical significance of our findings indicates the need for further research.

### Clinical implications

This study supports the assessment of PSE at the first physiotherapy appointment. High PSE is a predictor of a better outcome and low PSE is a predictor of a poorer outcome. This analysis suggests that manual therapy, electrotherapy, or acupuncture in addition to advice and exercise offer no improvement in pain or disability at six months, irrespective of PSE. Initial evidence suggests some patients who receive these treatments experience more pain and disability at six months compared to those who do not. However, as stated previously, the design of our study was not primarily for this research question, and we therefore recommend that these results provoke further discussion and as a basis for further research rather than be taken at face value.

Exercise therapy, in its many forms, is the gold standard physiotherapy treatment in the initial management of shoulder pain.^29–31^ Though the use of additional modalities such as manual therapy has been considered to reduce pain and improve disability in the short term,^32^ our results question the longer-term effects. We recommend that the harms, as well as potential benefits of passive interventions, be considered. We recommend interventions to improve PSE with respect to return to activity and participation, which may include, for example, behaviour change interventions.

### Research implications

Our results may be subject to confounding by indication. However, given the statistical significance of some of our findings, we recommend that future randomised controlled trials of passive physiotherapy interventions include measures of baseline pain, disability and PSE and further investigate this potential interaction. Stratifying study participants by baseline PSE, pain and disability could begin to explore how effective physiotherapy interventions are for those with differing baseline characteristics.

Future research is needed to further explore PSE as a treatment effect moderator for different physiotherapy interventions. PSE research has focused primarily on prognostic accuracy or to a smaller extent, test interventions designed to increase PSE.^33,34^ In addition to exploring interventions to treat PSE itself, further research is needed to investigate how clinicians can use PSE in a clinically meaningful way to best select interventions for optimal outcomes. This research could benefit patients through personalised or stratified physiotherapy treatments for musculoskeletal shoulder pain, for those with high and low PSE.

## Conclusion

This interactional analysis suggests that PSE does not moderate the effectiveness of manual therapy, acupuncture and/or electrotherapy with outcome. Compared to those not receiving these treatments, participants receiving manual therapy, acupuncture and/or electrotherapy in addition to advice and exercise, consistently had equal or worse mean pain and disability six months.

We advise caution on the interpretation of our results. Our study was not designed to investigate treatment effectiveness; participants were not randomly allocated to receive or not receive treatment, the decision for which is often complex and multifactorial.

We suggest future randomised controlled trials investigating these interventions include measures of harm as well as benefits and include measures of PSE to further investigate this potential interaction, which could in turn inform future personalised or stratified treatment of shoulder pain.

## Supplemental Material

sj-docx-1-sel-10.1177_17585732221105562 - Supplemental material for Shoulder pain: Is the outcome of manual therapy, acupuncture and electrotherapy different for people with high compared to low pain self-efficacy? An analysis of effect moderation Click here for additional data file. Supplemental material, sj-docx-1-sel-10.1177_17585732221105562 for Shoulder pain: Is the outcome of manual therapy, acupuncture and electrotherapy different for people with high compared to low pain self-efficacy? An analysis of effect moderation by Bradley Rugg, Mizanur Khondoker and Rachel Chester in Shoulder & Elbow

sj-docx-2-sel-10.1177_17585732221105562 - Supplemental material for Shoulder pain: Is the outcome of manual therapy, acupuncture and electrotherapy different for people with high compared to low pain self-efficacy? An analysis of effect moderation Click here for additional data file. Supplemental material, sj-docx-2-sel-10.1177_17585732221105562 for Shoulder pain: Is the outcome of manual therapy, acupuncture and electrotherapy different for people with high compared to low pain self-efficacy? An analysis of effect moderation by Bradley Rugg, Mizanur Khondoker and Rachel Chester in Shoulder & Elbow

sj-docx-3-sel-10.1177_17585732221105562 - Supplemental material for Shoulder pain: Is the outcome of manual therapy, acupuncture and electrotherapy different for people with high compared to low pain self-efficacy? An analysis of effect moderation Click here for additional data file. Supplemental material, sj-docx-3-sel-10.1177_17585732221105562 for Shoulder pain: Is the outcome of manual therapy, acupuncture and electrotherapy different for people with high compared to low pain self-efficacy? An analysis of effect moderation by Bradley Rugg, Mizanur Khondoker and Rachel Chester in Shoulder & Elbow

sj-docx-4-sel-10.1177_17585732221105562 - Supplemental material for Shoulder pain: Is the outcome of manual therapy, acupuncture and electrotherapy different for people with high compared to low pain self-efficacy? An analysis of effect moderation Click here for additional data file. Supplemental material, sj-docx-4-sel-10.1177_17585732221105562 for Shoulder pain: Is the outcome of manual therapy, acupuncture and electrotherapy different for people with high compared to low pain self-efficacy? An analysis of effect moderation by Bradley Rugg, Mizanur Khondoker and Rachel Chester in Shoulder & Elbow
