# Supplementary material for: Shoulder pain: Is the outcome of manual therapy, acupuncture and electrotherapy different for people with high compared to low pain self-efficacy? An analysis of effect moderation
Source: Shoulder Elbow. 2022 Jun 20;15(6):680–8. doi: 10.1177/17585732221105562 (PMC10656971; doi:10.1177/17585732221105562)

Supplementary file 1: Boxplot for the any manual therapy treatment category.

**6-month follow up SPADI scores for the any manual therapy category**


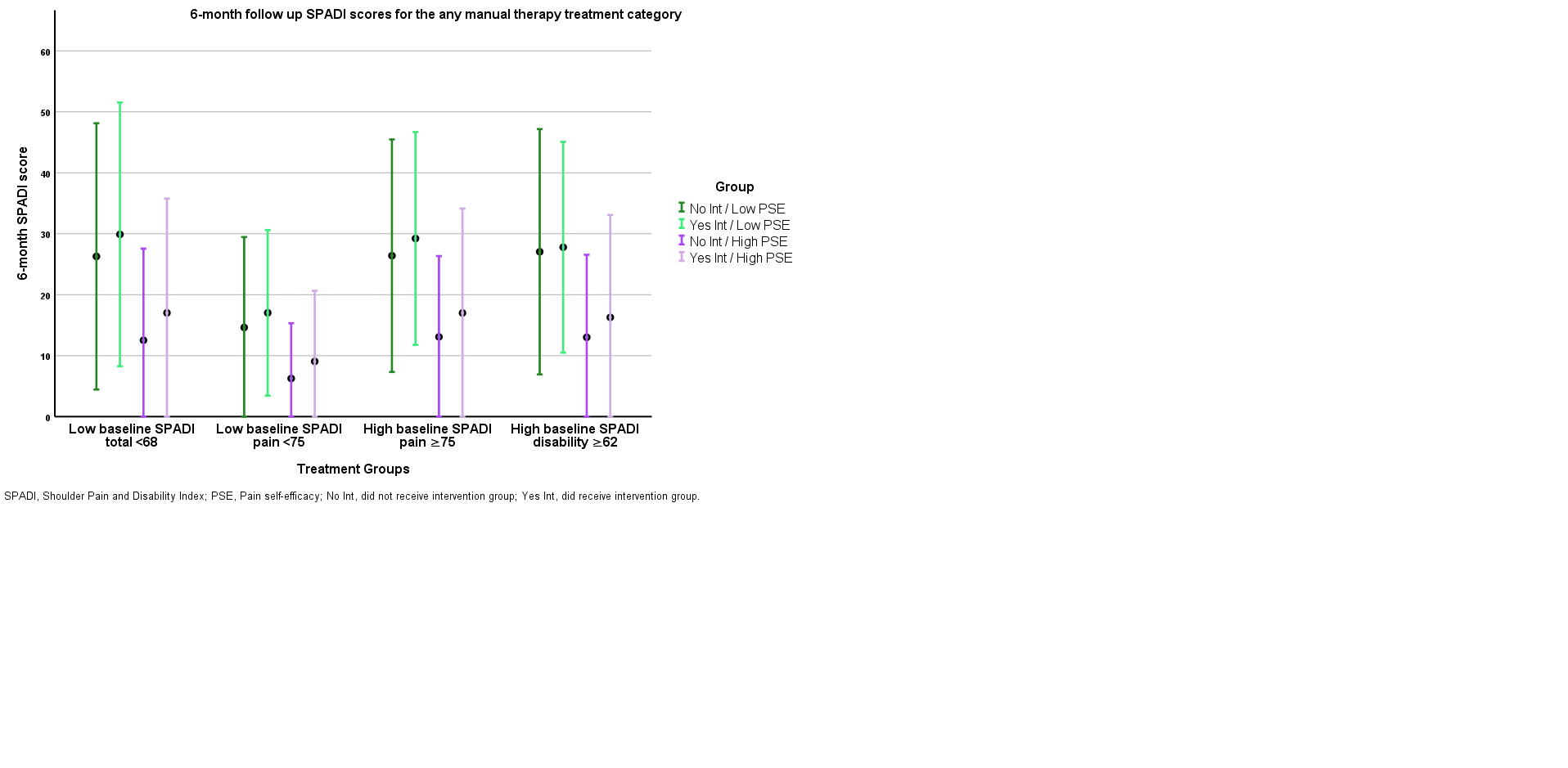

Supplement: sj-docx-1-sel-10.1177_17585732221105562 - Supplemental material for Shoulder pain: Is the outcome of manual therapy, acupuncture and electrotherapy different for people with high compared to low pain self-efficacy? An analysis of effect moderation [file sj-docx-1-sel-10.1177_17585732221105562.docx]
