# Supplementary material for: Shoulder pain: Is the outcome of manual therapy, acupuncture and electrotherapy different for people with high compared to low pain self-efficacy? An analysis of effect moderation
Source: Shoulder Elbow. 2022 Jun 20;15(6):680–8. doi: 10.1177/17585732221105562 (PMC10656971; doi:10.1177/17585732221105562)
Supplement: sj-docx-2-sel-10.1177_17585732221105562 - Supplemental material for Shoulder pain: Is the outcome of manual therapy, acupuncture and electrotherapy different for people with high compared to low pain self-efficacy? An analysis of effect moderation [file sj-docx-2-sel-10.1177_17585732221105562.docx]

Supplementary file 2: Results table for the any manual therapy category.

| Mean (+/- SD) SPADI subscores at six-month follow up, differences between treatment and PSE groups, and difference of difference (interaction) for the any manual therapy category. | | | | | |
| --- | --- | --- | --- | --- | --- |
|  |  | High PSE group | | Low PSE group | Difference between PSE groups |
| SPADI Subscore | | Mean ± SD (n) | | | Mean (95% CI) |
| **Low SPADI Total (<68)** | Received Treatment | 17.01 ± 18.76 (277) | 29.89 ± 21.64 (85) | | -12.88 (-17.36, -8.41) |
|  | Did not receive treatment | 12.53 ± 15.03 (205) | 26.28 ± 21.84 (57) | | -13.75 (-19.16, -8.35) |
| Difference between treatment groups Mean (95% CI) | | 4.48 (1.16, 7.81) | 3.61 (-2.56, 9.79) | | Difference of difference  0.87 (-6.14, 7.89) |
| **Low SPADI Pain (<75)** | Received Treatment | 9.06 ± 11.59 (277) | 17.03 ± 13.58 (92) | | -7.98 (-10.70, -5.25) |
|  | Did not receive treatment | 6.26 ± 9.06 (201) | 14.62 ± 14.84 (61) | | -8.37 (-11.68, -5.05) |
| Difference between treatment groups Mean (95% CI) | | 2.80 (0.70, 4.90) | 2.41 (-1.33, 6.15) | | Difference of difference  0.39 (-3.90, 4.68) |
| **High SPADI Pain (≥75)** | Received Treatment | 16.99 ± 17.14 (29) | 29.23 ± 17.45 (71) | | -12.24 (-19.85, -4.62) |
|  | Did not receive treatment | 13.08 ± 13.26 (20) | 26.4 ± 19.07 (53) | | -13.32 (-22.39, -4.25) |
| Difference between treatment groups Mean (95% CI) | | 3.91 (-6.13, 13.96) | 2.83 (-3.45, 9.10) | | Difference of difference  1.08 (-10.76, 12.93) |
| **High SPADI Disability (≥62)** | Received Treatment | 16.28 ± 16.81 (29) | 27.79 ± 17.29 (98) | | -11.51 (-18.92, -4.11) |
|  | Did not receive treatment | 13.01 ± 13.55 (19) | 27.04 ± 20.12 (56) | | -14.03 (-22.33, -4.73) |
| Difference between treatment groups Mean (95% CI) | | 3.27 (-7.07, 13.60) | 0.74 (-5.12, 6.61) | | Difference of difference  2.52 (-9.37, 14.41) |
| PSE, pain self-efficacy; SPADI, Shoulder Pain and Disability Index; SD, standard deviation; CI, confidence interval; n, number of participants. | | | | | |
