# Supplementary material for: Shoulder pain: Is the outcome of manual therapy, acupuncture and electrotherapy different for people with high compared to low pain self-efficacy? An analysis of effect moderation
Source: Shoulder Elbow. 2022 Jun 20;15(6):680–8. doi: 10.1177/17585732221105562 (PMC10656971; doi:10.1177/17585732221105562)
Supplement: sj-docx-4-sel-10.1177_17585732221105562 - Supplemental material for Shoulder pain: Is the outcome of manual therapy, acupuncture and electrotherapy different for people with high compared to low pain self-efficacy? An analysis of effect moderation [file sj-docx-4-sel-10.1177_17585732221105562.docx]

Supplementary file 4: Results table for the spinal/shoulder joint mobilisation category.

| Mean (+/- SD) SPADI subscores at six-month follow up, differences between treatment and PSE groups and difference of difference (interaction) for the spinal/shoulder joint mobilisation category. | | | | | |
| --- | --- | --- | --- | --- | --- |
|  |  | High PSE group | | Low PSE group | Difference between PSE groups |
| SPADI Subscore | | Mean ± SD (n) | | | Mean (95% CI) |
| **Low SPADI Total (<68)** | Received Treatment | 9.06 ± 11.31 (207) | 16.97 ± 13.71 (63) | | -7.91 (-11.03, -4.78) |
|  | Did not receive treatment | 6.76 ± 9.52 (275) | 14.34 ± 12.81 (79) | | -7.58 (-10.34, -4.81) |
| Difference between treatment groups Mean (95% CI) | | 2.30 (0.30, 4.29) | 2.63 (-1.04. 6.29) | | Difference of difference  -0.33 (-4.50, 3.84) |
| **Low SPADI Pain (<75)** | Received Treatment | 21.74 ± 22.09 (199) | 34.56 ± 25.22 (78) | | -12.83 (-18.57, -7.08) |
|  | Did not receive treatment | 17.01 ± 19.05 (270) | 32.71 ± 26.25 (84) | | -15.70 (-21.07, -10.33) |
| Difference between treatment groups Mean (95% CI) | | 4.72 (0.71, 8.74) | 1.85 (-4.91, 8.61) | | Difference of difference  2.87 (-4.99, 10.74) |
| **High SPADI Pain (≥75)** | Received Treatment | 37.27 ± 30.83 (22) | 55.27 ± 28.21 (49) | | -17.99 (-32.44, -3.54) |
|  | Did not receive treatment | 29.04 ± 25.14 (27) | 50.43 ± 29.16 (75) | | -21.39 (-34.03, -8.75) |
| Difference between treatment groups Mean (95% CI) | | 8.24 (-7.94, 24.41) | 4.84 (-5.51, 15.18) | | Difference of difference  3.40 (-15.80, 22.60) |
| **High SPADI Disability (≥62)** | Received Treatment | 14.12 ± 15.36 (20) | 27.97 ± 17.71 (75) | | -13.85 (-22.62, -5.07) |
|  | Did not receive treatment | 13.92 ± 13.36 (25) | 27.18 ± 19.23 (82) | | -13.26 (-21.23, -5.29) |
| Difference between treatment groups Mean (95% CI) | | 0.20 (-10.26, 10.66) | 0.79 (-4.78, 6.36) | | Difference of difference  -0.59 (-12.44, 11.27) |
| PSE, pain self-efficacy; SPADI, Shoulder Pain and Disability Index; SD, standard deviation; CI, confidence interval; n, number of participants. | | | | | |
